# Associations Between the Multidimensional Body Image Constructs and Self-Rated Health among African American Adults in the Southeastern United States: A Preliminary Study

**DOI:** 10.1007/s40615-025-02519-1

**Published:** 2025-06-23

**Authors:** Ingrid K. Richards Adams, James B. Odei, Kathy D. Wright, Wilson Figueroa, Michika R. Nickerson, Suzanne Leson, Oluwaseyi Agboola, Vedat O. Yildiz

**Affiliations:** 1 College of Food, Agricultural, and Environmental Sciences, The Ohio State University Extension, Columbus, OH 43201 USA; 2 Medical Dietetics, School of Health and Rehabilitation Sciences, The Ohio State University College of Medicine, 453 W. 10th Ave., Atwell Hall 306 D, Columbus, OH 43210 USA; 3 College of Public Health, The Ohio State University, 1841 Neil Avenue, 248 Cunz Hall, Columbus, OH 43210 USA; 4 College of Nursing, The Ohio State University, 376 Newton Hall, 295 W. 10th Avenue, Columbus, OH 43210 USA; 5 Comprehensive Cancer Center, The Ohio State University, 410 W 12th Ave, Columbus, OH 43210 USA; 6 Office of Nutrition, 25 South Front St., 3rd Floor, Columbus, OH USA; 7 School of Health and Rehabilitation Sciences, The Ohio State University College of Medicine, 453 W. 10th Ave, Columbus, OH 43210 USA; 8 Center for Biostatistics, College of Medicine, The Ohio State University, 320-37 Lincoln Tower, 1800 Cannon Drive, Columbus, OH 43210 USA

**Keywords:** African American adults 1, Body image 2, Obesity 3, African American males 4, African American females 5, Multidimensional Body Self-Reflection Questionnaire 6, Health disparity

## Abstract

**Background:**

This pilot study examined sex differences in body image constructs among African American (AAs) adults and their association with self-rated health (SRH).

**Methods:**

A cross-sectional study was conducted, where participants were recruited through advertisements and snowball sampling techniques. A battery of questionnaires measured demographic data, SRH, and body image constructs. Percent body fat and body mass index were collected using the BOD POD(R). Descriptive statistics, parametric tests, binary logistics, and linear regressions were performed with regression models adjusted for age, income, BMI, and education.

**Results:**

The majority (*n* = 54, 75.00%) of participants rated their health as good (40.28%) or very good/excellent (34.72%). Significant positive associations were observed between self-rated health, appearance evaluation, and appearance satisfaction. In univariate and multivariable models, for a one-unit increase in appearance evaluation score, participants had 3.85-fold (*p* = 0.003) and 3.71-fold (*p* = 0.037) odds of rating their health as good/excellent compared to rating their health as poor/fair, respectively. A significant negative association was observed between self-rated health and self-classified weight.

**Conclusion:**

Some aspects of body image constructs differed among African American males and females. Understanding these differences can help health professionals develop targeted interventions to promote positive health behaviors within this population. Mixed method studies are recommended to explore these differences further.

## Introduction

African American adults experience the highest age-adjusted prevalence of obesity (49.9%) compared to Hispanics (45.6%), Whites (41.4%), and Asian (16.1%) adults [1]. African American women bear a heavier brunt of this burden, with more than half (56.9%) classified as obese compared to 28.7% of White women and 31.2% of both White and African American men. [2–4].

The environments in which individuals live, learn, work, play, and worship directly influence their health. Factors of the environment, such as economic stability, education, health care access, and social and cultural contexts, contribute to a range of adverse health outcomes, including obesity, diabetes, cardiovascular disease, hypertension, and other chronic conditions [5–10]. African Americans experience higher rates of obesity and chronic disease primarily due to lower socioeconomic status, living in economically disadvantaged neighborhoods, reduced access to health care, higher levels of stress and discrimination, and nuanced social and cultural contexts that influence their behaviors [2, 11–13]. Among the social and cultural factors influencing health, body image perceptions may also serve as an important contributing factor to the obesity epidemic among African Americans. These perceptions can also impact their self-rated health, a key indicator of overall health and well-being [14, 15].

Body image is typically defined as the attitudes and perceptions individuals hold toward their bodies, including perceptual (estimation of body weight and size), cognitive-affective (attitudinal and cognitive feelings toward one’s body), and behavioral (self-checking behaviors) components [16]. While poor body image, body dissatisfaction, disordered eating, and poor mental health are associated with a higher body mass index (BMI) among non-Hispanic whites, research shows that African Americans may not be as vulnerable to the negative impact of body image as their non-Hispanic white counterparts [17–22]. On average, African Americans tend to have a more positive body image, i.e., a more favorable view of larger body sizes, fewer concerns about dieting, lower levels of fat anxiety, and less fluctuation in weight. They are also less likely to perceive themselves as overweight or obese compared to non-Hispanic whites [16, 21, 23]. Research suggests that African Americans are less likely to internalize Western ideals of beauty, which often prioritize thinness [24–27].

Despite existing literature, significant gaps remain in understanding body image and its relationship with obesity among African American adults. First, African Americans, particularly women, adopt a multidimensional definition of beauty and attractiveness that extends beyond slimness to include factors such as the fit of clothing, shapeliness, having defined hips, and femininity [28]. Current research mainly emphasizes a unidimensional view focused mainly on body dissatisfaction and its negative effect on health, neglecting the complexity of body image among African Americans [29–31]. Consequently, there is also a need for a shift to instruments that measure the multifaceted construct of body image of African Americans [32, 33].

Second, some studies suggest that differences regarding body image and size preference between non-Hispanic whites and African Americans–especially African American women–may be overstated. Some research reports varying levels of dissatisfaction among African Americans across different weight categories, indicating a need for further examination [20, 34–36]. While demographic factors such as age, income, education, and sex have been shown to influence body image, few studies have specifically examined how sex-based differences shape body image perceptions among African American populations [31, 37, 38].

Third, self-rated health, or the subjective rating of an individual’s overall health, is associated with obesity, health outcomes, and mortality rates among African Americans in nationally representative samples. Therefore, it is essential to consider this factor when examining the overall health of African Americans [39–41]. Research has shown associations between body image (both satisfaction and dissatisfaction) and self-rated health [42, 43]. However, few studies have explored the multidimensional constructs of body image and the potential associations with self-rated health among African American adults [43–45]. Understanding these factors can provide valuable insights into culturally appropriate strategies for promoting effective weight management outcomes in this population [2, 46, 47].

The objectives of this study were to (1) Investigate the multidimensional constructs of body image among African American males and females and (2) Assess the relationship between the multidimensional constructs of body image and self-rated health among African American males and females.

## Materials and Methods

### Study Sample and Design

This study is a secondary analysis of cross-sectional data from a parent study that examined the nutrition and health issues of African Americans residing in a metropolitan area of the Southeastern United States. Details of the parent study are described elsewhere [48]. In brief, participants were recruited through African American churches, newspaper advertisements, bulletin boards, and snowballing techniques, where individuals informed others in their social networks about the study. Participants were included in the study if they identified as African American, were between 18 and 74 years old, and resided in the metropolitan area of Lexington, Kentucky for at least one year. The Institutional Review Board of The University of the University of Kentucky approved the study procedures through an expedited review, and all participants provided written informed consent. Following data collection (*n* = 105), participants with missing data on percent body fat (*n* = 20) and constructs of body image (*n* = 13) were excluded. The missing data can be attributed to participants opting out of the assessment of percent body fat.

### Measures

Demographics and self-rated health. The REACH 2010 St. Louis Healthy Heart Survey (REACH) was used in the study [49]. The demographic questions (age, sex, income, and education) were adapted from the Behavioral Risk Factor Surveillance System [50, 51]. The other questions were derived from the Missouri Cardiovascular Disease Telephone Survey, which has also been tested for reliability and validity [52–54]. The REACH consisted of an 89-item, closed-ended, multiple-choice survey, some of which included skip logic.

#### Outcome

Self-rated Health. This measure was assessed using a single question: “Would you say that, in general, your health is…” Responses were scored on a scale of 1–5 (1 = poor, 2 = fair, 3 = good, 4 = very good, 5 = excellent), with higher scores indicating better health. Self-rated health was further aggregated into two categories: Poor/Fair (scores 1–2) or Good/Excellent (scores 3–5) for analysis.

#### Covariates

Percent Body Fat and Body Mass Index (BMI). Percent body fat is recognized as a more accurate predictor of diabetes, cardiovascular disease, and other chronic conditions compared to traditional BMI measures [55–57]. In this study, percent body fat was assessed using air displacement plethysmography (BOD POD), which determines body composition based on body density (BD), and air displacement in an enclosed chamber relative to total body volume. The Ortiz equation (percent body fat = [485 ÷ Db] – 439) for females, and the Schutte equation (percent body fat = [437.7 ÷ Db] – 392.8) was used to calculate body fat for males [58, 59]. Individuals were classified as high (males ≥ 23%, females ≥ 32%) or low (males < 23%, females < 32%) percent body fat [60].

BMI was calculated using the standardized equation: (weight in kg)/(height in m)^2^ and participants were assigned categories of normal (18.5 to less than 25), overweight (25 to less than 30), and obese (30 or greater) [61].

Body Image. Body image was assessed using the Multidimensional Body-Self Relations Questionnaire [33], which included five constructs: (1) appearance evaluation, (2) appearance orientation, (3) appearance satisfaction, (4) overweight preoccupation, and (5) self-classified weight. All constructs, except appearance satisfaction, were scored on a scale of 1 to 5 (1 = definitely disagree and 5 = definitely agree). Appearance satisfaction was scored from 1 to 5 (1 = very dissatisfied to 5 = very satisfied), and self-classified weight was scored from 1 (very underweight) to 5 (very overweight). Appearance evaluation is a 7-item measure that assesses feelings of physical attractiveness or unattractiveness, as well as satisfaction or dissatisfaction with one’s appearance. Higher scores reflect mostly positive feelings and greater satisfaction with one’s appearance [62]. The calculated internal reliability for the current study was α = 0.79. Appearance orientation is a 9-item measure that evaluates cognitive and behavioral attention to appearance-related issues. It assesses the extent to which a person is invested in his/her appearance. Higher appearance satisfaction scores reflect greater emphasis or investment in one’s physical appearance [62]. The calculated internal reliability for the current study was α = 0.80. Appearance satisfaction, also referred to as the Body Areas Satisfaction Scale, assesses overall satisfaction with various areas of one’s body (i.e., face, hair, torso, muscle tone, weight, height, and overall appearance). Higher scores indicate satisfaction, while lower scores suggest dissatisfaction with different aspects of one’s body. The calculated internal reliability for the current study was α = 0.82. Overweight preoccupation measures concerns related to fat anxiety, weight vigilance, dieting, and eating restraint. Higher scores indicate a greater preoccupation with being/becoming overweight. The calculated internal reliability for the current study was α = 0.72. Finally, Self-classified weight measures individuals’ perception of their weight in relation to how they believe others would classify them, as well as one’s own classification of weight, from very underweight (1) to very overweight (5). Higher scores indicate a self-classification of being overweight, while lower scores indicate a self-classification of being underweight. The calculated internal reliability for the current study was α = 0.82.

### Conceptual Framework

**Fig. 1:**
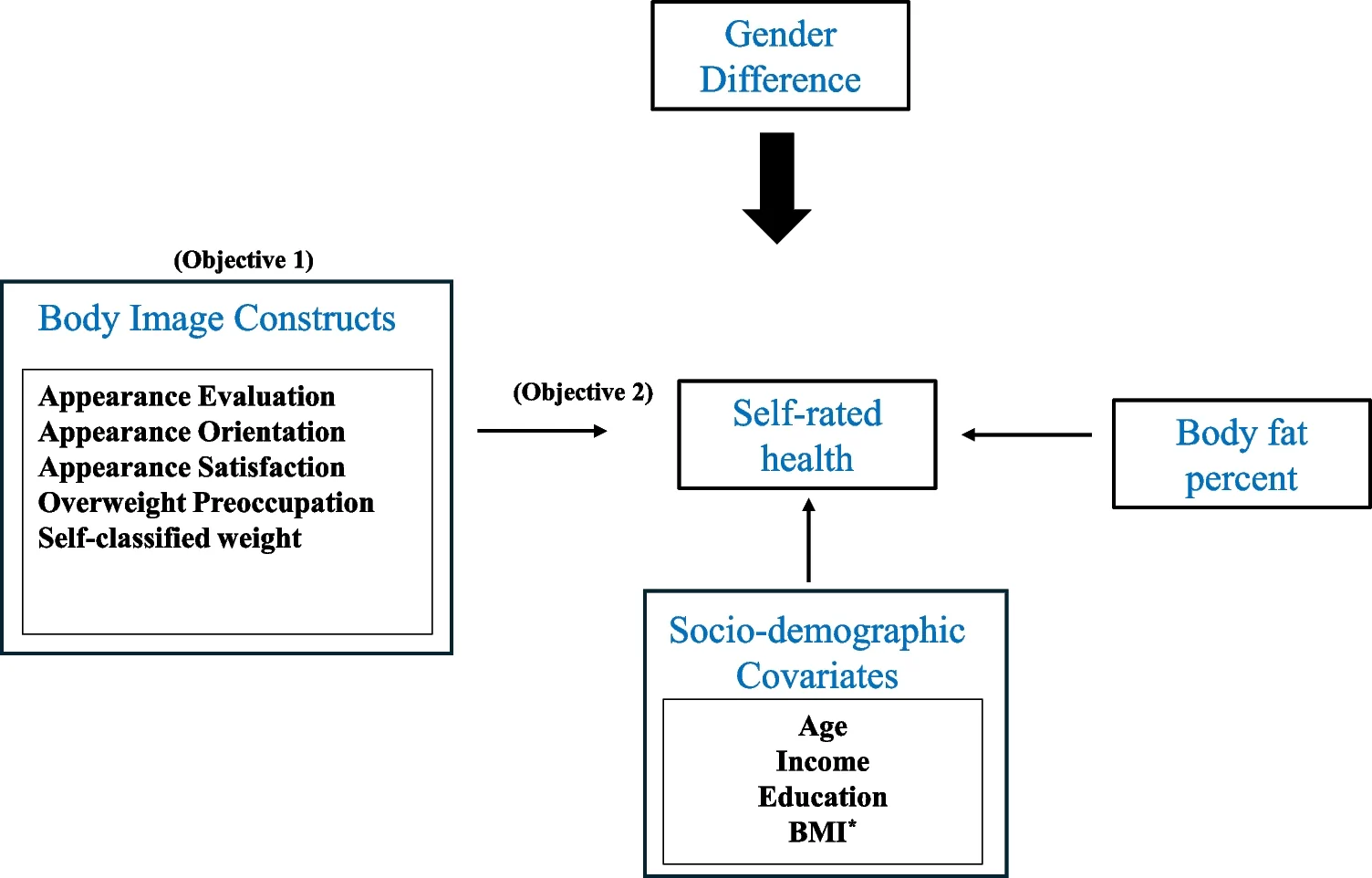
Conceptual Framework of the Associations between the multidimensional body image constructs and self-rated health among African American adults in the Southeastern United States. ^*^BMI was not included in the multivariate model due to collinearity with body fat percentage

The conceptual framework visually represents the key variables and concepts, as well as the presumed relationships among them. It emphasizes the importance of using a multidimensional construct of body image to capture the complex nature of body image among African Americans. The primary outcome in the framework is self-rated health, which reflects an individual’s overall perception of their health status. The various body image constructs are hypothesized to be associated with self-rated health, with each dimension potentially exerting a different influence on how African Americans perceive their health. Moreover, these influences may vary by gender, highlighting differences in how body image relates to self-rated health among males and females. Together, this framework offers a model for understanding how body image shapes health perceptions in African Americans, while acknowledging the moderating role of gender and the impact of other demographic factors. See Fig. 1.

### Statistical Analysis

Demographic data were analyzed to characterize the sample. Descriptive statistics were used to compare the baseline characteristics of the final cohort, using appropriate parametric tests for continuous variables (t-tests or Mann–Whitney tests) and the Chi-squared or Fisher’s exact tests for categorical variables. Participants self-rated their general health. However, due to smaller counts for some categories, self-rated health was further aggregated for analysis to Poor/Fair or Good/Excellent. The five constructs of body image were used in the analysis. Binary logistic regression models were used to examine the cross-sectional continuous association of the five constructs of body image with self-rated health. Each of these analyses were performed separately utilizing the total analytical cohort (n = 72). In each adjusted model, we controlled for age, sex, percent body fat, income, and education. Odds ratios (OR 95% confidence interval [CI]) were estimated for each body image construct and the outcomes) and interpreted as odds of self-rated health of Good/Excellent compared to Poor/Fair in the unadjusted and adjusted models. We didn’t include BMI in the multivariable model due to collinearity with percent body fat. A linear regression model was also used to examine the relationship between self-rated health and percent body fat. Here, in the adjusted model, we controlled for age, sex, income, and education. Additionally, for both the logistic and linear regression models, the association between the outcome and the covariate(s) was stratified by sex to examine differences between males and females. All analyses were performed using SAS software (version 9.4, SAS Institute Inc., Cary, NC, USA). Statistical significance was defined as a two-sided *p*-value < 0.05 for all analyses.

## Results

### Participant Characteristics

The final sample consists of 72 participants, comprising 56 females and 16 males. The overall characteristics of the participants, as well as those stratified by sex (male and female) and self-rated categories (poor/fair and good/excellent), are presented in Table 1. The majority of participants were female (77.8%), between the ages of 18 and 35 (55.6%), with some college experience or a college degree (66.2%), and had an income of less than $35,000 (68.7%). The cohort's mean age was 36.75 years (SD = 15.91). Most women (~ 80%) possessed high body fat. The mean percent body fat was 36.06% (SD = 12.54), with males differing significantly from females (25.00 vs. 39.22, *p* = < 0.0001). Concerning BMI, ~ 75% of males and ~ 73% of females fell within the overweight or obese categories.

**Table 1:** Characteristics of study participants by sex and self-rated general health

		Sex			General Health		
Baseline Characteristics	All	Male	Female	*p*-value	Poor/Fair	Good/very good/Excellent	p-value
Participants—*n* (%)	72 (100)	16 (22.2)	56 (77.8)		18 (25.0)	54 (75.0)	
Age—*n* (%)				.478			.660
18–35	40 (55.6)	8 (50.0)	32 (57.1)		10 (55.5)	30 (55.5)	
36–50	15 (20.8)	5 (31.2)	10 (17.9)		5 (27.8)	10 (18.5)	
> 50	17 (23.6)	3 (18.8)	14 (25.0)		3 (16.7)	14 (25.9)	
Age—mean ± SD [Range]	36.75 ± 15.91 [18.00–83.00]	36.88 ± 12.83 [19.00–54.00]	36.71 ± 16.79 [18.00–83.00]	.971	36.56 ± 13.35 [18.00–57.00]	36.81 ± 16.79 [18.00–83.00]	.952
Income—n (%)				.602			.679
$15,000	20 (29.9)	5 (31.2)	15 (29.4)		6 (33.3)	14 (28.6)	
$15,000- < $35,000	26 (38.8)	5 (31.2)	21 (41.1)		6 (33.3)	20 (40.8)	
$35,000- < $50,000	10 (14.9)	4 (25.0)	6 (11.8)		4 (22.2)	6 (12.2)	
$50,000	11 (16.4)	2 (12.6)	9 (17.7)		2 (11.2)	9 (18.3)	
Education—n (%)				.932			.357
High School	24 (33.8)	6 (37.5)	18 (32.7)		8 (47.0)	16 (29.6)	
Some College	33 (46.5)	7 (43.8)	26 (47.3)		7 (41.2)	26 (48.5)	
College Graduate	14 (19.7)	3 (18.7)	11 (20.0)		2 (11.8)	12 (22.2)	
Percent Body Fat—*n* (%)				.0002*			.236
Low^1^	22 (30.6)	11 (68.8)	11 (19.7)		3 (16.7)	19 (35.2)	
High^2^	50 (69.4)	5 (31.2)	45 (80.3)		15 (83.3)	35 (64.8)	
Percent Body Fat—mean ± SD [Range]	36.06 ± 12.54 [10.51–62.07]	25.00 ± 11.30 [10.51–50.91]	39.22 ± 11.06 [10.97–62.07]	<.0001*	40.74 ± 12.51 [11.33–56.10]	34.50 ± 12.26 [10.51–62.07]	.066
BMI—*n* (%)				.937			.244
Normal^3^	19 (26.4)	4 (25.0)	15 (26.8)		2 (11.1)	17 (31.5)	
Overweight^4^	24 (33.3)	6 (37.5)	18 (32.1)		7 (38.9)	17 (31.5)	
Obese^5^	29 (40.3)	6 (37.5)	23 (41.1)		9 (50.0)	20 (37.0)	
Body Image – mean ± SD [Range]							
Appearance Evaluation^6^	3.58 ± 0.72 [2.14–5.00]	3.74 ± 0.68 [2.43–5.00]	3.53 ± 0.73 [2.14–5.00]	.297	3.13 ± 0.67 [2.43–4.57]	3.72 ± 0.67 [2.14–5.00]	.001*
Appearance Orientation^7^	3.71 ± 0.55 [2.17–4.75]	3.60 ± 0.47 [2.92–4.33]	3.74 ± 0.56 [2.17–4.75]	.395	3.67 ± 0.54 [2.50–4.67]	3.72 ± 0.55 [2.17–4.75]	.749
Appearance Satisfaction^8^	3.56 ± 0.68 [2.11–5.00]	3.68 ± 0.63 [2.56–4.78]	3.53 ± 0.70 [2.11–5.00]	.429	3.02 ± 0.66 [2.11–4.78]	3.74 ± 0.60 [2.56–5.00]	<.0001*
Overweight Preoccupation^9^	2.37 ± 0.92 [1.00–4.50]	1.77 ± 0.76 [1.00–3.00]	2.54 ± 0.89 [1.00–4.50]	.002*	2.58 ± 0.94 [1.00–4.50]	2.30 ± 0.91 [1.00–4.00]	.269
Self-classified weight^10^	3.47 ± 0.80 [2.00–5.00]	3.22 ± 0.73 [2.00–4.50]	3.54 ± 0.82 [2.00–5.00]	.154	3.97 ± 0.93 [2.00–5.00]	3.31 ± 0.69 [2.00–5.00]	.001*
General Health—n (%)				.745			
Poor/Fair	18 (25.0)	3 (18.8)	15 (26.8)				
Good/very good/Excellent	54 (75.0)	13 (81.2)	41 (73.2)				

*p*-values calculated using chi-square or Fisher’s exact test (categorical variables), and t-test (parametric continuous variables); Range is reported as [Min–Max]; 1. Percent Body Fat low (males < 23%, females < 32%); 2. Percent Body Fat high (males ≥ 23%, females ≥ 32%); 3. BMI of 18.5 to less than 25; 4. BMI of 25 to less than 30; 5. BMI of 30 or greater; 6. Appearance evaluation refers to feelings of physical attractiveness; 7. Appearance orientation: Extent of investment in one’s appearance; 8. Appearance satisfaction: Overall satisfaction with various areas of one’s body (e.g., face, torso, hair); 9. Overweight preoccupation: Fat anxiety, weight vigilance, dieting, and eating restraint; 10. Self-classified weight: Self-perceived (how one thinks others would classify them) as well as one’s own classification of weight

^*^p <.05

Males scored higher in appearance evaluation (M = 3.74 [SD = 0.68] vs M = 3.53 [SD = 0.73]) and appearance satisfaction (M = 3.68 [SD = 0.63] vs M = 3.53 [SD = 0.70]) than females. Females scored higher than males in appearance orientation (M = 3.74 [SD = 0.56] vs M = 3.60 [SD = 0.47]), self-classified weight (M = 3.54 [SD = 0.82] vs M = 3.22 [SD = 0.73]), and overweight preoccupation (M = 2.54 [SD = 0.89] vs M = 1.77 [SD = 0.76]). These findings were only statistically different by sex for overweight preoccupation (p = 0.002).

There were no significant differences between how males and females rated their health. See Table 1 for further details.

### Association of Constructs of Body Image with Self-Rated Health

Table 2 presents the association between body image constructs and self-rated health. A significant positive association was observed between self-rated health and appearance evaluation (one’s feelings of physical attractiveness), appearance satisfaction (satisfaction with different body areas), and self-classified weight. In the unadjusted and adjusted models (i.e., after adjusting for age, sex, income, percent body fat and education status), for a unit increase in appearance evaluation score, the odds of self-reporting good/very good/excellent health versus poor/fair health among participants were 3.85-fold (p = 0.004) and 3.71-fold (p = 0.037) higher, respectively. In the unadjusted and adjusted models, for a unit increase in appearance satisfaction score, the odds of self-reporting good/excellent health versus poor/fair health among participants were 6.48-fold (p = 0.0005) and 14.04-fold (p = 0.002) higher, respectively. A significant negative association was observed between self-rated health and self-classified weight. In the unadjusted and adjusted models, a unit increase in self-classified weight was associated with a 69% (OR = 0.31, 95% CI: 0.14, 0.69) and 80% (OR = 0.20, 95% CI: 0.05, 0.83) lower odds of self-reporting good/excellent health versus poor/fair health, respectively. There was no significant association between self-rated health and appearance orientation, or overweight preoccupation (all p > 0.05) (Table 2).

**Table 2:** Odds ratios of self-rated general health by body image construct

Body Image Construct	Unadjusted Model OR (95% CI)	Adjusted Model^a^ OR (95% CI)
Appearance Evaluation	**3.85 (1.54, 9.62)** ***p* =.004**	**3.71 (1.07, 12.82)** ***p* =.037**
Appearance Satisfaction	**6.48 (2.25, 18.64)** ***p* =.0005**	**14.04 (2.52, 78.26)** ***p* =.002**
Appearance Orientation	1.17 (0.44, 3.13) *p* =.745	0.95 (0.27, 3.28) *p* =.934
Overweight Preoccupation	0.71 (0.39, 1.28) *p* =.259	0.77 (0.34, 1.74) *p* =.536
Self-classified Weight	**0.31 (0.14, 0.69)** ***p* =.004**	**0.20 (0.05, 0.83)** ***p* =.027**

*OR*: Odds Ratio, *CI*: Confidence Interval, p: *p*-value

*Statistically significant findings are in bold

^a^Model adjusted for age, sex, percent body fat, income, and education

Participants who rated their health as good/very good/excellent had 6.24% and 5.88% (unadjusted and adjusted, respectively) lower body fat percentage when compared to participants who self-reported their health as poor/fair (p = 0.066 and p = 0.044, respectively) (Table 3).

**Table 3:** The association of self-rated general health with body fat percentage

Body Fat %—Linear Regression – Beta Coef. (95% CI), *p*-value		
	Unadjusted Model (*n* = 72)	Adjusted Model^a^ (*n* = 72)
Poor/Fair	Referent	Referent
Good/very good/Excellent	‒6.24 (‒12.93, 0.44), *p* = 0.066	‒**5.88 (**‒**11.62, **‒**0.14), *p* = 0.044**

*CI* Confidence Interval, *p* *p*-value

*Statistically significant findings are in bold

^a^Model adjusted for age, sex, income, and education

### Association of Constructs of Body Image with Self-Rated Health Stratified by Sex

Table 4 presents the association between self-rated health and the body image constructs, stratified by sex. Among females, in the unadjusted and adjusted model, for a unit increase in appearance evaluation score, the odds of self-reporting good/excellent health versus poor/fair health among participants were 3.37-fold (OR = 3.7, 95% CI: 1.25, 9.08) and 3.05-fold (OR = 3.05, 95% CI: 0.83, 11.23) higher, respectively. This association was not significant in the multivariable analysis, after adjustment for age, income, and education.

**Table 4:** Odds ratios of self-rated general health by body image construct stratified by sex*

Body Image Construct	Unadjusted Model OR (95% CI)	Adjusted Model^a^ OR (95% CI)
**Appearance Evaluation**		
Female	**3.37 (1.25, 9.08)**	3.05 (0.83, 11.23)
Male	6.76 (0.61, 74.04)	n/a
**Appearance Satisfaction**		
Female	**7.93 (2.29 27.46)**	**25.33 (2.72, 235.32)**
Male	2.87 (0.32, 25.54)	n/a
**Appearance Orientation**		
Female	1.09 (0.38, 3.12)	0.77 (0.20, 2.97)
Male	2.79 (0.13, 60.08)	n/a
**Overweight Preoccupation**		
Female	0.80 (0.41, 1.58)	0.86 (0.37, 2.03)
Male	0.40 (0.07 2.33)	n/a
**Self-classified Weight**		
Female	**0.32 (0.13, 0.76)**	**0.11 (0.01, 0.80)**
Male	0.33 (0.05, 2.15)	n/a

*OR* Odds Ratio, *CI* Confidence Interval, *p* *p*-value, *Statistically significant findings are in bold

^a^Model adjusted for age, percent body fat, income, and education

Although this association was non-significant for males, the odds of self-reporting good/excellent health versus poor/fair health was 6.76-fold (unadjusted model) higher per unit increase in appearance evaluation score. Performing the multivariable analysis for the male sample regarding body image construct variables was not feasible due to the limited sample size. Regarding appearance satisfaction, among females, the odds of self-reporting good or excellent health compared to poor or fair health were 7.93-fold and 25.33-fold higher, respectively, in the unadjusted and adjusted models.

Also, in the adjusted model, a unit increase in appearance orientation was associated with 23% lower odds of self-reporting good/excellent health compared to poor/fair health, for females. However, this association was not statistically significant. Additionally, we found a significant negative association between self-rated health and self-classified weight for females, (OR = 0.11, CI: 0.01, 0.80). There was no significant association between overweight preoccupation and self-classified weight by sex.

Females with self-rated health as good/excellent had 4.74% and 5.26% (unadjusted and adjusted, respectively) lower body fat percentage when compared to females who self-reported their health as poor/fair (Table 5). Similarly, males with self-rated health as good/excellent had 7.40% and 10.82% (unadjusted and adjusted, respectively) lower body fat percentage when compared to males who self-reported their health as poor/fair (Table 5). However, these associations were not statistically significant.

**Table 5:** The association of self-rated general health with body fat percentage by sex

Body Fat %—Linear Regression – Beta Coef. (95% CI), *p*-value		
	**Female**	
	Unadjusted Model (*n* = 56)	Adjusted Model^a^ (*n* = 50)
Poor/Fair	Referent	Referent
Good/Excellent	‒4.74 (−11.37, 1.88), *p* = 0.157	‒5.26 (‒11.57, 1.04), *p* = 0.099
	**Male**	
	Unadjusted Model (*n* = 16)	Adjusted Model^a^ (*n* = 16)
Poor/Fair	Referent	Referent
Good/very good/Excellent	‒7.40 (‒22.89, 8.08), *p* = 0.322	‒10.82 (‒26.37, 4.72), *p* = 0.147

*CI* Confidence Interval, *p* *p*-value

^a^Model adjusted for age, income, and education

## Discussion

This exploratory study examined the multidimensional constructs of body image among African American males and females, exploring the relationship between the various body image constructs and self-rated health. Results showed most participants had high body fat percentages, with over two-thirds of males and nearly three-quarters of females classified as overweight or obese. Among both males and females, a significant positive association was observed between appearance evaluation and appearance satisfaction. Negative associations were found between self-classified weight and both body fat percentage and self-rated health. Among females, a significant positive association was observed between appearance satisfaction, and a significant negative association was seen between self-classified weight and self-rated health.

The study identified sex differences in the multidimensional constructs of body image. For example, females scored numerically higher in appearance orientation, self-classified weight, and overweight preoccupation. In contrast, males showed numerically higher scores in appearance evaluation and appearance satisfaction, which encompassed feelings of attractiveness and satisfaction towards various body parts, including the face, hair, torso, muscle tone, weight, and height. These results, though not statistically significant, are aligned with previous research. For instance, Smith et al. examined body image (appearance evaluation and appearance orientation) in a population-based, biracial cohort consisting of African Americans (n = 3,768) as part of a multi-year study–the Coronary Artery Risk Development in Young Adults Study [62]. Their results showed that African American women invested more time in managing their appearance (appearance orientation) than their male counterparts. This focus on appearance among African American women may stem from a broader definition of beauty that encompasses skin tone, hair, and facial features. Awad et al. noted that, while often overlooked, hair is a significant aspect of beauty and body image for African American women, with many investing considerable time and resources in hair care. Consequently, this focus on appearance may require greater attention to detail, leading to a larger time commitment for women. [63, 64]. Smith et al. similarly showed that African American males reported higher satisfaction with their physical attractiveness than African American females. This difference may be attributed to lower obesity rates among males, contributing to discrepancies in appearance satisfaction and evaluation between genders [62].

Fitzgibbon et al. found that black women reported dissatisfaction only when they were overweight or obese. Given that approximately three-quarters of females in this study were overweight and obese, this may help explain the observed gender differences in overweight preoccupation [65].

A positive correlation was observed between self-rated health and both appearance evaluation and appearance satisfaction for both males and females. The odds of self-reporting good/very good/excellent health compared to poor/fair health were 6.33-fold higher in the unadjusted model and 15.33-fold higher in the adjusted model. Studies have shown that across all racial groups, higher levels of perceived attractiveness (as evaluated by appearance) correlate with better self-rated health. The National Longitudinal Study of Adolescent to Adult Health (N = 13,638) found that both males and females who rated themselves as more attractive also reported better self-rated health [45]. Additionally, Williamson et al. explored health- and appearance-related concerns among young African American and Caucasian females using the Multidimensional Body-Self Relations Questionnaire [66]. They found that satisfaction with appearance was more closely linked to self-rated health among African American women. Subtle nuances between African males and females in appearance evaluation and self-rated health among African Americans were also observed. For example, men who perceived themselves as only slightly attractive still rated their health positively, while women had to perceive themselves as at least moderately attractive before they reported good health. While self-rated health is subjective, it correlates with objective dimensions of health and serves as a strong predictor of morbidity and mortality [67]. It is important, first of all, that African Americans be aware and be informed that feelings of attractiveness cannot be equated with general health. It should be emphasized that health screenings and yearly exams should be pursued in spite of how a person feels or looks, as appearance satisfaction does not necessarily reflect objective health status. Future research is needed to explore the factors associated with the multidimensional constructs of body image and self-rated health among African Americans [41].

A significant positive association was observed between self-rated health and self-classified weight among African American females. In both unadjusted and adjusted models, a unit increase in self-classified weight was associated with 56% and 63% lower odds of reporting good or excellent health compared to poor or fair health, respectively. While some studies suggest that African Americans, especially females, show greater acceptance of body weight and sizes, leading to inaction towards improving their health or engaging in weight management strategies [68–70], findings from this study suggest otherwise. Participants expressed dissatisfaction with larger body sizes, a desire to lose weight, and reported poor self-rated health at higher BMIs. Hastings used a representative national sample to examine the associations between self-rated health and obesity among a diverse group of Black individuals (African Americans and Caribbean males and females). Results showed that having obesity was linked to poor self-rated health among all groups and genders [15]. However, research by Agyemang and Powell-Wiley showed that African Americans are less likely to receive lifestyle modification counseling among healthcare providers [71]. Patient-provider communication is an important aspect of treatment adherence, successful obesity self-management, and health outcomes for African Americans. Thus, it is essential for providers to address weight-related issues in a culturally appropriate manner. For example, Di Nola et al. recommend a patient-centered approach that considers individual views on their ideal body image [72].

This study has several limitations that must be acknowledged. First, the overall sample size is small, particularly for males, which can impact the robustness of the findings and their association with effect size [73]. The use of a cross-sectional design and a convenience sampling strategy makes it difficult to make causal inferences and is susceptible to non-response and recall biases [74]. Additionally, regional characteristics and socio-cultural views may influence the association between body image and self-rated health, thus limiting the generalization of the study. For example, perception of body image may differ among African Americans in different areas of the US. Despite these limitations, this study provides valuable preliminary evidence highlighting the differences in body image constructs among African American males and females, as well as the potential association between these differences and self-rated health. By examining these nuances, the research lays the groundwork for future research on body image and self-rated health within the African American population.

## Conclusions

Few studies have thoroughly examined the within-group differences of multidimensional body image constructs among African Americans, with a focus on sex-specific differences. This study reveals some significant variations in body image constructs between African American males and females. Understanding these differences can help health professionals develop targeted interventions to promote positive health behaviors in this population. Weight management strategies should be tailored to address the unique needs of African American men and women. The observed positive associations between self-rated health and appearance evaluation emphasize the need for culturally appropriate interventions that distinguish between feelings of physical attractiveness and overall health. Given the higher odds among females, it is crucial to target this group, especially as they experience higher rates of obesity compared to other ethnic groups. Another important conclusion from this study is that dissatisfaction with body image and weight varies significantly among African Americans, particularly females. Healthcare professionals must recognize these disparities and address weight-related concerns in a culturally appropriate manner. Finally, it is important to reemphasize that while body image represents an important social and cultural factor influencing obesity among African Americans, it does not operate in isolation. Research shows that it is part of a broader context of social, cultural, and structural determinants, which include socioeconomic status, access to healthcare, neighborhood environments, and systemic discrimination, that drive disparities in obesity and chronic disease.

Future research should explore the diverse dimensions of body image, its development, and its associations with self-reported health in both African American males and females. Employing mixed methods approaches would allow for a deeper understanding of individual experiences, offering rich data and new insights that are often overlooked when relying solely on quantitative methodologies. Such explorations are important in developing culturally responsive and relevant strategies for reducing health disparities.

## Data Availability

Data supporting Tables 1, 2, 3, 4 and 5 are available upon request by contacting the corresponding author.
